# Green Extraction from Pomegranate Marcs for the Production of Functional Foods and Cosmetics

**DOI:** 10.3390/ph9040063

**Published:** 2016-10-18

**Authors:** Raffaella Boggia, Federica Turrini, Carla Villa, Chiara Lacapra, Paola Zunin, Brunella Parodi

**Affiliations:** Department of Pharmacy, University of Genoa, Viale Cembrano 4, I-16148 Genoa, Italy; turrini@difar.unige.it (F.T.); villa@difar.unige.it (C.V.); lacapra@difar.unige.it (C.L.); zunin@difar.unige.it (P.Z.); parodi@difar.unige.it (B.P.)

**Keywords:** waste valorization, ultrasound-assisted extraction (UAE), microwave-assisted extraction (MAE), enriched foods and cosmetics

## Abstract

The aim of this study was to investigate the potential of retrieving polyphenolic antioxidants directly from wet pomegranate marcs: the fresh by-products obtained after pomegranate juice processing. These by-products mainly consist of internal membranes (endocarp) and aril residues. Even if they are still edible, they are usually discharged during juice production and, thus, they represent a great challenge in an eco-sustainable industrial context. Green technologies, such as ultrasound assisted extraction (UAE) and microwave assisted extraction (MAE), have been employed to convert these organic residues into recycled products with high added value. UAE and MAE were used both in parallel and in series in order to make a comparison and to ensure exhaustive extractions, respectively. Water, as an environmentally friendly extraction solvent, has been employed. The results were compared with those ones coming from a conventional extraction. The most promising extract, in terms of total polyphenol yield and radical scavenging activity, has been tested both as a potential natural additive and as a functional ingredient after its incorporation in a real food model and in a real cosmetic matrix, respectively. This study represents a proposal to the agro-alimentary sector given the general need of environmental “responsible care”.

## 1. Introduction

Even if pomegranate is one of the oldest known edible fruits, only recently has it recaptured consumers’ interest, as it has gained significant popularity in the last few years as a functional food and nutraceutical source, becoming a high value crop.

Pomegranate fruit is currently considered as one of the new superfoods. In the last 10 years the number of scientific publications on pomegranate and its health benefits have greatly increased. Promising results have been reported in the treatment of cardiovascular disease, diabetes, and prostate cancer [1]. These activities have been attributed to the phenolic fraction of pomegranate [2]. Anthocyanins and hydrolysable tannins, especially ellagitannins (such as punicalagin, punicalin, pedunculagin, and the aglycone ellagic acid) are present in high concentration in pomegranate-based products and they are responsible for their high antioxidant activity [3,4,5]. This evidence indicates that compounds extracted from pomegranate could be used as multipurpose bioactive ingredients [6].

In general, there are several methods to obtain extracts from plant products. Traditionally the conventional methods involve the use of chemical solvents. However, it is known that they represent a problem related to waste disposal and environmental impact. In addition, some of them are not food-grade solvents; thus, it is further necessary to find an exhaustive purification step during food production in order to avoid residual toxicity.

Green procedures, namely those complying with standards set by Environmental Protection Agency of the USA (http://www.epa.gov/greenchem-istry/pubs/about_gc.html), have several advantages, compared to conventional solid-liquid extraction techniques, such as the reduction of extraction times and energy consumption, chemical solvent removal, extraction efficiency, safety, and high extract quality [7,8,9,10,11].

In microwave assisted extraction (MAE), the microwave energy is delivered directly to materials through molecular interactions with the electromagnetic field via conversions of electromagnetic energy into thermal energy [12]. The process acceleration and high extraction yield may be the result of a synergistic combination of two transport phenomena: heat and mass gradients working in the same direction [13]. On the other hand, in conventional extractions the mass transfer occurs from inside to outside, although the heat transfer occurs from outside to inside of the substrate. In addition, although in conventional extraction the heat is transferred from the heating medium to the interior of the sample, in MAE the heat is dissipated volumetrically inside the irradiated medium. With the help of microwaves, extractions can be completed in minutes instead of hours with several advantages (e.g., high reproducibility, less solvent and energy consumption, more compact procedures, and enhanced purity of the final product).

Recently, ultrasound-assisted extraction (UAE) of foodstuff has received significant interest, since it is relatively low-cost. It overcomes the disadvantages of traditional solvent extractions and, at the same time, it provides higher quantities of extracts in the same unit of time, using smaller amounts of food-grade solvent or just water [14]. Ultrasounds are able to mechanically break the cell walls extracting the intracellular liquids. The ultrasound waves (kHz range) act on the molecules of a liquid medium, namely the solvent, by alternating moments of compression and moments of rarefaction on the solvent molecules. These loops of compression and expansion cycles cause the formation of voids or air bubbles that gradually grow until implosion occurs, giving rise to the phenomenon of cavitation. UAE can be used both on a small and large scale in the food extraction industry.

The aim of this study was to investigate the potential of retrieving polyphenolic antioxidants by green extractions directly from wet pomegranate juice marcs, the fresh edible by-products obtained after pomegranate juice processing. These by-products consist mainly of internal carpellary membranes (endocarp) and of aril residues (including seeds) that remain after juice pressing, since in the classic production of pomegranate juice, exocarp (rind) and mesocarp (white spongy tissue) are preliminarily separated to avoid excessive turbidity and astringency of the juice itself. Even if the non-edible pomegranate peels (exocarp and mesocarp) are widely investigated in literature [15,16,17,18,19,20], the edible discarded part left over from the production of pomegranate juice (here named "pulp") is another waste that could be valorized, since coming from the edible part it is free of regulatory constraints. Membrane and aril residues are characterized by the presence of hydrolysable tannins, phenolic acids (i.e. ellagic acid and gallic acid), and flavonoids that can be retrieved for nutraceutical and cosmeceutical applications [21].

In order to evaluate the functional properties of these extracts in a real food matrix and in a real cosmetic formulation, the most promising extract was used to enrich fresh-cut apple slices, as a model food, and to enrich a cosmetic hydrophilic gel, as a model cosmetic.

## 2. Results and Discussion

### 2.1. Green Extraction

**Scheme 1:**
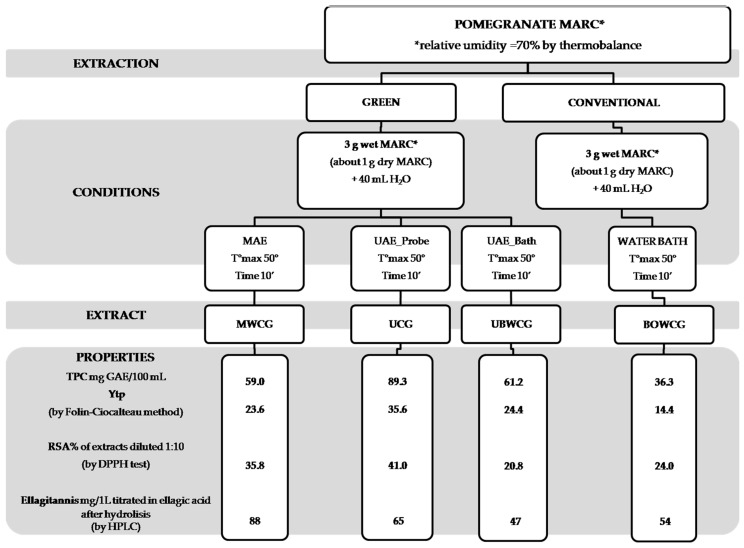
The different extraction techniques used in parallel, the process conditions, the corresponding extracts’ names, and properties are reported, respectively.

**Scheme 2:**
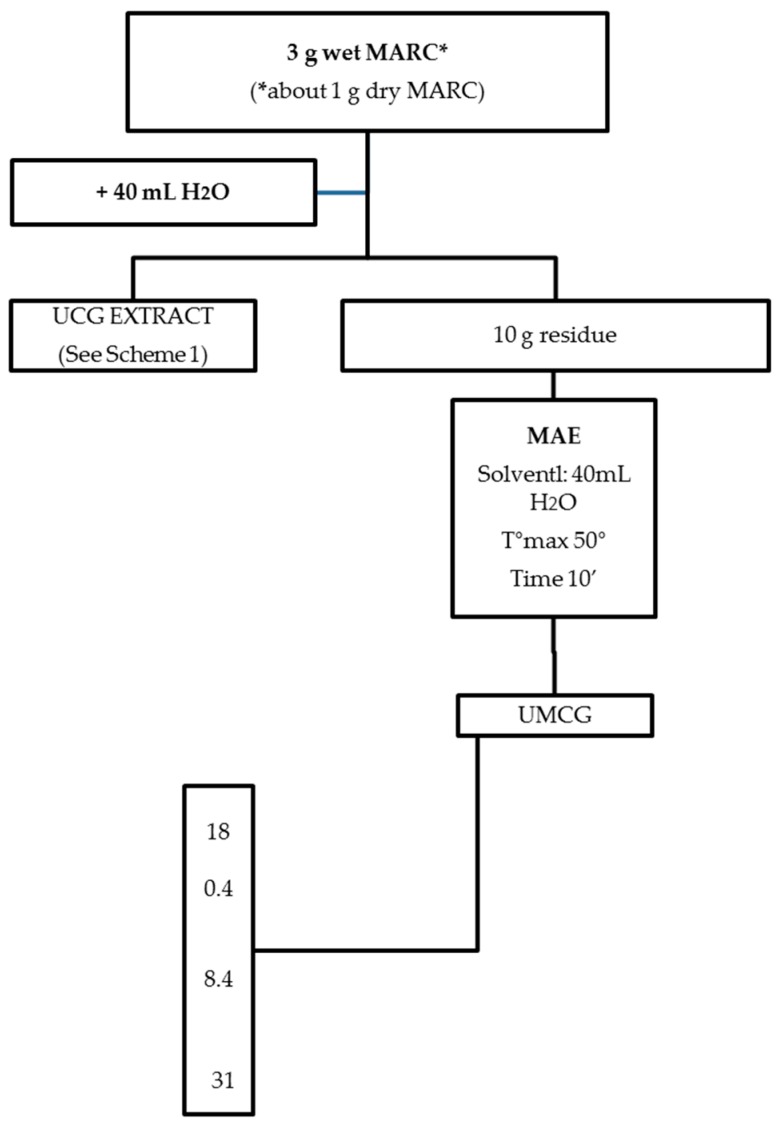
UAE and MAE used in series, the process conditions, the corresponding extracts’ names, and properties are reported, respectively.

Green extraction technologies, such as microwave assisted extraction (MAE) and ultrasound assisted extraction (UAE), were both used in parallel (Scheme 1), in the same operating conditions to compare the different techniques, and in series, in order to ensure exhaustive extractions (Scheme 2). A conventional extraction, whose heating was achieved with a water bath, was performed in order to compare the green technologies with a traditional one in the same operating conditions. All of the extraction techniques were performed using just deionized water as the solvent. The corresponding extracts were evaluated and compared on the basis of several properties, such as the total phenolic contents (expressed both as TPC, total phenolic compounds content, in mg GAE/100 mL of extract and as Y_tp_, total polyphenols yield, in mg GAE/g of marc), the total amount of ellagitannis, which are the predominant class of phytochemicals of pomegranate fruits, and the radical scavenging ability (RSA).

The results of all the extractions and the corresponding extracts properties are reported in Scheme 1 and Scheme 2.

As far as TPC and RSA are concerned, each assay was replicated three times from the pulp extraction to the property quantification, and the statistical significance was determined using V-PARVUS 2010 (University of Genova, Genova, Italy) [22] and the Excel Data Analysis Tool (Microsoft Corporation, Seattle, WA, US). The descriptive statistics are reported in Table 1 and Table 2.

The results were analyzed using one way analysis of variance (ANOVA) for comparison between groups followed by paired-group comparisons by Student’s *t*-test. In particular, one-way ANOVA was used to analyze the mean differences among the extracts obtained using all of the different technologies, while Student’s *t*-test was used to analyze differences between the extract obtained by each green extraction and the conventional ones.

ANOVA analysis showed significant differences (*p* < 0.05) between total phenolic compounds (F = 610.150, dof = 3, Fcrit = 4.066, *p*-value = 8.80837 × 10^−10^) and radical scavenging activity (F = 136.157, dof = 3, Fcrit = 4.066, *p*-value = 0.00000033) of all of the resulting extracts.

Student’s *t*-test showed that all the green extracts are significantly different (*p* < 0.05) from the conventional ones in term of TPC and RSA.

As far as the parallel extractions are concerned both MAE and UAE performed with a sonotrode produce two aqueous extracts (labeled in Scheme 1 MWCG and UCG respectively) with a good compromise among polyphenolic content (TPC) and yield (Yt), ellagitannins content, and RSA. Nevertheless, UCG is slightly better than MWCG, above all in terms of RSA, thus UCG was taken into account for further investigation. Its residue (first extraction residue) was further extracted by microwave in order to verify if the first UAE extraction was sufficiently exhaustive (Scheme 2).

Scheme 2 reports the details of this second extraction and the properties of the second extract (labeled UMCG). The low values of UMCG properties showed that the second extractive step is useless since, evidently, the first extraction had been exhaustive.

UCG, the most promising extract, has been compared with the pomegranate fruit itself in terms of antioxidant potential. TPC and Trolox equivalent antioxidant capacity (TEAC) of the juice have been quantified as 462 mg GAE/ 100 mL and 54.2 mM, respectively. TPC and TEAC of UCG have been quantified as 89 mg GAE/ 100 mL and 4.1 mM, respectively. The UCG values are lower than those of the corresponding juice, but comparable with those of many other common fruit juices as reported by Phenol-Explorer database (http://phenol-explorer.eu/).

### 2.2. Enrichment of Food and Cosmetics

In order to evaluate the functional properties of the green extracts both in a real food matrix and in a real cosmetic formulation, the most promising extract was used to enrich, respectively, fresh-cut apple slices, as the model food, and cosmetic hydrophilic gel, as the model cosmetic.

As far as foodstuff is concerned, UCG has been tested in providing protection against oxidative browning in apple slices (1–1.5 cm) after five minutes of dipping in this extract and in a comparable standard solution of gallic acid (in term of GAE = gallic acid equivalents) using water as a control. 

**Figure 1:**
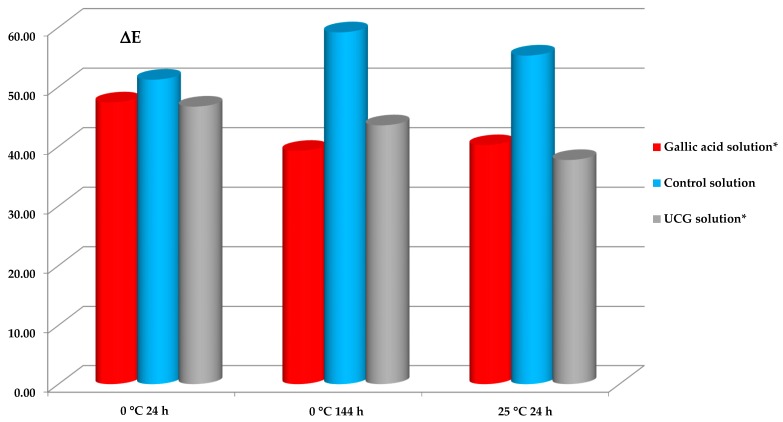
Total color difference (ΔE) to evaluate the anti-browning potential of the solutions. * Solution with the same concentration in terms of GAE.

Total color differences (ΔE) of the apple slices with respect to an initial time (t_0_) were used to evaluate the anti-browning potential of the solutions after dipping the apple slices. Two storage times, after 24 h (both at 4 °C and 25 °C) and after 144 h (0 °C), were taken into account, respectively. Since an increase of ΔE with respect to the initial time means the occurrence of browning, ΔE is inversely correlated with the anti-browning potential. The results are summarized in Figure 1, where the efficiency of UCG extract in inhibiting the enzymatic browning of a model food is highlighted. The behavior of UCG with respect to a control dipping solution, made by water, is comparable to that one of the gallic acid solution. This evidence confirms the results obtained by the direct analysis of the extracts also once used in a real food matrix. Moreover the presence of UCG in the minimally-processed fresh-cut apples gives them the connotation of “nutraceutical foods” delivering the bioactive molecules of pomegranate edible marc without the offensive astringency typical of the rind extracts.

As far as cosmetics are concerned, UCG was used and evaluated as a functional antiradical ingredient in a cosmetic hydrophilic gel containing hyaluronic acid, xanthan gum, glycerol, and UCG 20%. The samples were also compared either to a gel containing a comparable amount of gallic acid (in term of GAE = gallic acid equivalents) and to a non-enriched gel, as a control. The antiradical activity (RSA) of each hydrogel is reported in Table 3. The results are in accordance with those obtained by the direct analysis of the extracts despite of the complexity of the matrices. Even if the RSA of the UCG enriched hydrogel is lower than the gallic acid enriched one, it is higher than the non-enriched hydrogel used as a control. Once again this preliminary result confirms the antioxidant potential of UCG also after incorporation in a real matrix, such as a cosmetic formulation.

## 3. Materials and Methods

### 3.1. Chemicals

All chemicals were purchased from Sigma-Aldrich (Steinheim, Germany) and from VVR Chemicals (Radnor, PA, USA). High purity water (HPW) produced with a Millipore Milli-Q system (Millipore, Bedford, MA, USA) was used throughout. All solvents used for chromatographic purposes were HPLC grade.

### 3.2. Plant Material

About 10 kg of ripened pomegranate fruits (*Punica granatum* cv. Dente di Cavallo) were collected at ripening in the second week of October (maturity index, calculated as total soluble solids (TSS)/titratable acidity (TA) = 33.2) from a small producer located in Calabria region, Southern Italy (province of Reggio Calabria, 38°6′41″40 N, 15°39′43″56 E).

The following morphological traits were observed: fruits of medium–large size (200–250 g) and spherical shaped, with a semi-opened calix and a yellowish-red thin rind. The interior of the fruits had thin carpellary membranes, the arils were pink and prismatic of medium size with hard seeds.

The fruits were stored at room temperature for a few days until used. Then they were rinsed with distilled water, manually peeled and processed by a commercial pomegranate juice processor (Borz Srl, Rovereto, Italy). The corresponding juice was of red color and had a sweet taste. The juice yield was about 53%, the edible by-products and the non-edible by-products have been about 22% and 24% respectively. The total soluble solids content (TSS) of the juice was 14.6° Brix, determined by a digital refractometer at 20 °C (Hanna Instruments, Padova, Italy), the pH was 4.11, measured using a pH meter (Jenway, Staffordshire, UK), and the titratable acidity (TA) was 0.44%, determined by titration to pH 8.1 with 0.1 M NaOH solution and expressed as g of citric acid per 100 g of juice. The average maturity index of the fruits was 33.2, calculated as TSS/TA. The wet pomegranate juice marcs were collected and, after the relative humidity determination, were stored at −80 °C until further use. All measurements were made in triplicate and the average results reported.

### 3.3. Green Extraction

Green extraction technologies, such as microwave-assisted extraction (MAE) and ultrasound-assisted extraction (UAE), were used both in parallel (Scheme 1), and in series (Scheme 2). Water, as an environmentally friendly extraction solvent, has been employed to treat the pomegranate pulp by-products. Both the green extractions and the conventional one were performed for 10 min, keeping the temperature under control, always below 50 °C, starting from 3.0 g of wet pomegranate pulp by-product, corresponding to about 1 g of dry weight, and 40 mL of deionized water in order to have a 1:40 solid:solvent ratio.

The obtained extraction mixtures were filtered on paper and centrifuged (3000 rpm for 10 min), and then they were named as reported in Scheme 1 and Scheme 2 and stored at –20 °C until further analysis.

#### 3.3.1. Microwave Assisted Extraction (MAE)

The experimental apparatus for microwave irradiation was a single mode scientific reactor (Discover®, CEM Corporation, Matthews, NC, USA) with temperature feedback control, temperature monitoring by an optical fiber sensor, and a maximum output power of 300 watts. Water was used as the solvent for extraction and all extraction experiments were replicated three times. As far as process conditions are concerned, the same ones of UAE were chosen in order to make an easier comparison, after some preliminary trials in order to verify that those conditions comprised a good range of operating conditions.

#### 3.3.2. Ultrasound Assisted Extraction (UAE)

Ultrasonic extraction was performed directly and indirectly using, respectively:

- a sonicator with an operating frequency of 26 kH, effective output of 200 watts, equipped with a titanium (7 mm i.d.) sonotrode (Hielscher Ultrasonics UP200 St, Teltow, Germany); and
- an ultrasonic bath (BandelinSonorex RK52, Berlin, Germany) with 35 kHz frequency, maximum power of 240 watts, and internal dimension of 150 × 140 × 100 mm.

Process conditions of the UAEs were determined using already published results [23,24] after several preliminary trials to adapt them. The details are reported in Table 4.

The results were compared with a conventional extraction using a conventional water bath. The conventional extraction was performed by soaking 3.0 g of wet pomegranate marc with the same amount of water used for the green extraction (40 mL of deionized water) under steady stirring while maintaining a constant temperature of 50 °C for 10 min.

### 3.4. Determinations

#### 3.4.1. Determination of the Relative Humidity of the Pomegranate Marcs

The moisture content (relative humidity) of the wet pomegranate juice marcs were determined to be 70.0 ± 2.1 by a Sartorius moisture analyzer (Sartorius AG, Goettingen, Germany). All measurements were made in triplicate and the average results reported.

#### 3.4.2. Determination of Total Phenolic Compound Content Using the Folin–Ciocalteu Method

To determine the amount of total phenolic content (TPC) in the extracts, the Folin–Ciocalteu methodology [25] using gallic acid as the reference standard, was employed. Briefly, in a test tube, 0.2 mL of a sample appropriately diluted, 1 mL of diluted Folin–Ciocalteu reagent (diluted 1:10 with deionized water), 0.8 mL of aqueous sodium carbonate 7.5% *w/v* were added and vortexed. Then the mixture was allowed to stand at room temperature in the dark (25 ± 2 °C), for 30 min. The absorbance was read at 760 nm in an Agilent 8453 UV-VIS spectrophotometer (Agilent Technologies, Paolo Alto, USA) with 1 nm resolution, and the total polyphenol concentration was calculated from a calibration curve, using gallic acid as a standard.

The content of total phenolic compounds was expressed as milligrams of gallic acid equivalent (GAE) in 100 mL of the extract.

Then, the total polyphenol yield (Y_tp_) was calculated as follows:

Y_tp_ (mg GAE/g) = (mg GAE/ml × V)/m
GAE = mg gallic acid equivalents of the corresponding extract
V = volume of the extraction medium (mL)
m = the dry weight of the pulp (g)

#### 3.4.3. Determination of Radical Scavenging Activity (RSA) Using the DPPH Method

To determine the radical scavenging activity (RSA) of the extracts, one of the most commonly used in vitro assays, namely the DPPH (2,2-diphenyl-1-pycrylhydrazyl) was performed [26,27]. Aliquots (0.250 mL) of the extracts were transferred into a 10 mL volumetric flask and a daily prepared DPPH mother solution (approximately 10^−4^ M in methanol) was added. The reaction flask was kept in the dark for 30 min. The residual absorbance was measured by an Agilent 8453 UV-VIS spectrophotometer at 515 nm, at 25 °C. Blank (solution without radical) was measured before each sample and subtracted. The initial DPPH concentration was measured by control samples (without extracts and named “control”), obtained by diluting 0.250 mL of methanol with the DPPH mother solution in a 10 mL volumetric flask. The RSA of the samples were expressed as the % reduction of DPPH concentration in a DPPH solution exactly 1.00 × 10^−4^ M and was not dependent on the concentration of the daily DPPH solutions:

RSA (%) = ([DPPH] _control_ − [DPPH] _sample_)/10^−4^ × 100

Alternatively, absorbance measurements are transformed in antioxidant activity using 6-hydroxy-2,5,7,8-tetramethyl-2-carboxylic acid (Trolox) as a reference. Results are expressed as Trolox equivalent antioxidant capacity (TEAC).

#### 3.4.4. Determination of Ellagic Acid (EA) Content by HPLC

A rapid HPLC method was employed to quantify both the free (FEA) and the total ellagic (TEA) acid contents. TEA was obtained after acid hydrolysis of the ellagitannins present in the extracts. HPLC and hydrolysis were carried out as described by Huerga-González et al. in 2015 [28], with slight modifications. Briefly, the acid hydrolysis was obtained by adding 2 mL of 2 M HCl to 2 mL of each extract and heating at 100 °C for 1 h. After cooling, 1 mL of 2 M NaOH and 6 mL of methanol were added to the vial and injected into the chromatograph after filtration through a 0.50-μm filter.

An HP1100 (Agilent Technologies, Paolo Alto, USA) liquid chromatographic system equipped with a diode array detector (DAD) was used. The column was a Kromasil 100-5-C18 (Akzo-Nobel, Amsterdam, NL) (250 × 4 mm, 5-μm particle size) and was thermostated at 30 °C. The mobile phase was (A) acetic acid/methanol/water (2:400:598) and (B) methanol, with a flow rate of 1.0 mL/min, starting from 100% A. At 25 min 100% B was linearly reached, and then held for 10 min. The column was then reported back to the initial conditions in 5 min and the system was re-equilibrated for 5 min. Spectra were recorded between 220 and 600 nm by DAD. EA was identified by its chromatographic behavior and UV spectrum and its content was determined at 245 nm by the external standard method (EA standard solutions in methanol). The coefficient of variation was estimated equal to 7.3% by repeating the same analysis three times on the same sample, starting from the pulp extraction to the property quantification.

### 3.5. Enrichment of a Cosmetic Hydrophilic Gel

A bioactive hydrogel composed by sodium hyaluronate (1%), xanthan gum (1%), glycerol (10%), UCG (20%), and water, was prepared. UCG and glycerol were dissolved in about 78 ml of water in a beaker. Then sodium hyaluronate and xanthan gum were added and dispersed under stirring and gentle warming (40 °C). In order to make a comparison, a gel composed by sodium hyaluronate (1%), xanthan gum (1%), glycerol (10%), and a solution of gallic acid (comparable to UCG in terms of GAE = gallic acid equivalents) 20% was also prepared, as already mentioned. A non-enriched gel, containing sodium hyaluronate (1%), xanthan gum (1%), glycerol (10%), and water was used as a control. All of the prepared gels were finally stored in an opportune vessel at ambient temperature. until analysis, performed on 0.250 mL of each gel by the above mentioned DPPH test in order to evaluate the RSA.

### 3.6. Enrichment of Fresh-Cut Apple Slices

Whole biodynamic apples (cv. Golden) of a high maturity grade were a gift of a local Italian producer (TERRE DI FRUTTA Az. Agr. Bunino, Cavour, Turin, Italy).The fruits were cleaned, peeled, cored, and cut into 1–1.5 cm thick slices. The apple slices were, respectively, dipped for five minutes in the following three solutions: UCG extract, a standard solution of gallic acid whose concentration was equivalent to the GAE content of UGC, and water as a control solution. After drainage of the excess of solutions, the apple slices were immediately analyzed (initial time, t_0_) by a Cary 100 UV-VIS double beam spectrophotometer (Varian Cary 100, Agilent Technologies, Paolo Alto, CA, USA). Then, two storage times (t): after 24 h (both at 4 °C and 25 °C) and after 144 h (0 °C), were taken into account, respectively.

### 3.7. Color Analysis

UV-VIS analysis was applied in diffuse reflectance (DR) mode to study the influences of the extracts as anti-browning agents on fresh cut Golden apple slices as model foods. UV-VIS analysis was carried out using a Cary 100 UV-VIS double beam spectrophotometer equipped with a DRA (Diffuse Reflectance Accessory) integrating sphere and with a solid sample holder. The reference used was a white disk filled with compressed Spectralon^®^ powder. Spectra were collected in the 360–830 nm range at a resolution of 1 nm. Three replicates of each sample were recorded and the average signals were taken into account. The instrumental setup allowed diffuse reflectance to be measured, thus, the specular component of the reflected light was excluded. The CIELAB coordinates: L^∗^ (lightness), *a*^∗^ (reddish–greenish), and *b*^∗^ (yellowish–bluish) of the apple slices were automatically calculated from the spectral data by the Cary 100 WinUV color software using the CIE D65 illuminant.

In order to evaluate the anti-browning potential of the tested dipping solutions/extract, the total color differences (ΔE) were calculated matching the enriched apple slices spectrum at the initial storage (t_0_) and its relative spectrum at one of the two already mentioned storage times (t), according the following equation:

$$\Delta E=\sqrt{{\left({L^{*}}_{t}-{L^{*}}_{t_{0}}\right)}^{2}+{\left({a^{*}}_{t}-{a^{*}}_{t_{0}}\right)}^{2}+{\left({b^{*}}_{t}-{b^{*}}_{t_{0}}\right)}^{2}}$$

## 4. Conclusions

Many studies published in the scientific literature affirm that the pomegranate bioactive molecules can exert numerous healthy effects preventing, or even slowing, several diseases’ progression. Ellagitannins and, in particular, their gut microbial metabolites [29] seem to be the most important class of phytochemicals involved in the nutraceutical properties of pomegranate. These polyphenols are still significantly present in the pomegranate by-products after pomegranate juice extraction. In particular, the by-products coming from the inner part of the fruit, even if less rich in polyphenols, are edible and their taste is more pleasant and less astringent with respect to that coming from the outer part. In the present study several green extraction techniques are proposed to valorize this by-product by turning an industrial waste into a valuable ingredient for foods and cosmetics. The conversion from organic residues to recycled products with high added values represents a great challenge in an eco-sustainable industrial context, due both to the general need of environmental “responsible care” and to the need in the agro-alimentary sector to treat a large amount of organic residues that are often quite expensive to be disposed of or are quite pollutant. 

In particular, one of the extracts obtained by UAE is demonstrated to be a high added-value product. It is a useful ingredient by virtue of its anti-browning activity, as a potential natural additive, and a functional ingredient by virtue of its antioxidant properties, valuable even after its incorporation in a real food model and/or in a real cosmetic matrix.

Moreover, the green extraction, with respect to conventional extraction, have led to an improvement in terms of extraction yields using the same process conditions in terms of temperature, time, and solvent. Finally, the use of water as an environmentally friendly solvent is another important added value of the proposed techniques, since the protection of the environment from toxic materials, in particular from petroleum-based solvents, is one of the most important performances of a process in terms of cost, safety, and health issues. 

## Figures and Tables

**Table 1:** Folin–Ciocalteu results of parallel extractions.

	MWCG	UCG	UBWCG	BOWCG
Total polyphenols (TPC) mg GAE/100mL(Mean)	59	89	61	36
Standard error of the mean (SEM)	0.91	0.74	0.74	1.08
Standard deviation	1.58	1.28	1.28	1.87
Variance	2.48	1.65	1.63	3.50
Confidence level (95.0%)	3.92	3.19	3.17	4.65

**Table 2:** 2,2-Diphenyl-1-pycrylhydrazyl (DPPH) test results of parallel extractions.

	MWCG	UCG	UBWCG	BOWCG
RSA% of theextracts diluted 1:10 (Mean)	36	41	21	24
Standard error of the mean (SEM)	0.96	0.92	0.79	0.54
Standard deviation	1.66	1.60	1.36	0.93
Variance	2.76	2.56	1.86	0.87
Confidence level (95.0%)	4.13	3.98	3.39	2.31

**Table 3:** DPPH test results of the enriched cosmetic gel.

	RSA%
Cosmetic gel with 20% gallic acid solution *	37
Cosmetic gel with 20% UCG solution *	13
Control cosmetic gel	0

* Solution with the same concentration in terms of GAE.

**Table 4:** UAE process condition details.

Direct UAE (Sonotrode)	Conditions	Indirect UAE (Bath)	Conditions
Pulsed mode: duty cycle per second (DC)	50%		
Solvent	Deionized Water	Solvent	Deionized Water
Dry pomegranate marc/solvent ratio	1:40	Dry pomegranate marc/solvent ratio	1:40
Amplitude level	40%		
Extraction time	10 min.	Extraction time	10 min.
Extraction Temperature	<50 °C	Extraction Temperature	<50 °C
